# Mapping of lithium ion concentrations in 3D structures through development of *in situ* correlative imaging of X-ray Compton scattering-computed tomography

**DOI:** 10.1107/S1600577524003382

**Published:** 2024-06-05

**Authors:** Chu Lun Alex Leung, Matthew D. Wilson, Thomas Connolley, Chun Huang

**Affiliations:** a Department of Mechanical Engineering University College London LondonWC1E 7JE United Kingdom; b Research Complex at Harwell Rutherford Appleton Laboratory DidcotOX11 0FA United Kingdom; c STFC-UKRI Rutherford Appleton Laboratory DidcotOX11 0QX United Kingdom; d Diamond Light Source DidcotOX11 0QX United Kingdom; e Department of Materials Imperial College London LondonSW7 2AZ United Kingdom; f The Faraday Institution DidcotOX11 0RA United Kingdom; University of Malaga, Spain

**Keywords:** X-ray Compton scattering computed tomography, XCS-CT, microstructures, ion concentrations, ion transport, tomography

## Abstract

Understanding the correlation between chemical and microstructural properties is critical for unraveling the fundamental relationship between materials chemistry and physical structures that can benefit materials science and engineering. Here, we demonstrate novel *in situ* correlative imaging of the X-ray Compton scattering computed tomography (XCS-CT) technique for studying this fundamental relationship. XCS-CT can image light elements that do not usually exhibit strong signals using other X-ray characterization techniques. This paper describes the XCS-CT setup and data analysis method for calculating the valence electron momentum density and lithium-ion concentration, and provides two examples of spatially and temporally resolved chemical properties inside batteries in 3D. XCS-CT was applied to study two types of rechargeable lithium batteries in standard coin cell casings: (1) a lithium-ion battery containing a cathode of bespoke microstructure and liquid electrolyte, and (2) a solid-state battery containing a solid-polymer electrolyte. The XCS-CT technique is beneficial to a wide variety of materials and systems to map chemical composition changes in 3D structures.

## Abstract 2

A novel *in situ* correlative imaging X-ray Compton scattering computed tomography technique has been developed to unravel the fundamental relationship between the materials chemistry of light elements and their 3D structures. Here, this technique is applied to two types of batteries to map the internal lithium-ion concentrations.

## 1. Introduction

X-ray computed tomography (XCT) has been used for studying internal structures of materials during solidification (Withers *et al.*, 2021 ▸), battery operation (Huang *et al.*, 2021 ▸), additive manufacturing (Leung *et al.*, 2019 ▸) and many other processes (Karagadde *et al.*, 2021 ▸). Using high-flux synchrotron X-rays, the acquisition time of XCT has sped up from a matter of minutes to milliseconds (García–Moreno *et al.*, 2021 ▸; Leung *et al.*, 2022 ▸), capturing dynamic processes close to real time in most situations (Mokso *et al.*, 2017 ▸; van Aarle *et al.*, 2016 ▸). Tomoscopy is a technique that pushes temporal resolution down to just 1 ms (1000 tomograms per second are acquired) while maintaining spatial resolutions of micrometres and running experiments for minutes without interruptions (García–Moreno *et al.*, 2021 ▸; Leung *et al.*, 2022 ▸).

Rechargeable batteries are critical for electric transportation and storing electrical energy generated from intermittent renewable sources for net-zero applications. Among them, lithium-ion batteries are the current battery of choice, although newer types of batteries are also being actively developed (Huang *et al.*, 2017 ▸). During battery charging, lithium ions move from the cathode to the anode, and this process is reversed during discharging; reversible redox reactions between lithium and the electrode materials take place during charging and discharging.

XCT has been used to image battery components, for example, synchrotron XCT has been used to image crack growth through a ceramic solid-state electrolyte (SSE) pellet during charging inside a solid-state battery (Ning *et al.*, 2023 ▸). However, it is challenging to image changes in lithium concentration using XCT only, because lithium is one of the lightest elements and it has a low X-ray attenuation coefficient compared with the other transition metal elements inside batteries. Changes in X-ray attenuation due to varying lithium concentrations in battery cells are generally too low to yield sufficient contrast in conventional absorption-contrast imaging. To overcome this challenge, there is a need for developing a characterization technique that complements XCT.

It is also extremely challenging for other characterization techniques to measure lithium concentrations. For example, X-ray diffraction (XRD) is used for studying the crystal structure and lattice spacing of materials (Connolley *et al.*, 2020 ▸; Matras *et al.*, 2020 ▸). Although XRD can provide structural information about crystalline materials, it is not easy to perform characterization studies on amorphous materials or materials without long-range order (Chen *et al.*, 2024 ▸; Chen & Huang, 2023 ▸; Huang *et al.*, 2018 ▸). The pair distribution function (PDF) utilizes total scattering (Bragg and diffuse scattering) and has been used to investigate long-range disordered materials with short-range ordering (Wiaderek *et al.*, 2013 ▸). X-ray absorption spectroscopy (XAS) and X-ray near-edge absorption spectroscopy (XANES) can study the change of the oxidation state of elements (Yu *et al.*, 2014 ▸), but the signals from lithium are weak because there are fewer electron orbitals, characteristic edges or peaks in a lithium atom compared with other elements (McBreen & Balasubramanian, 2002 ▸; Spence *et al.*, 2021 ▸). The weak signals from lithium are often masked by strong signals from other heavy elements (*e.g.* transition metals) inside batteries. Soft X-ray scanning transmission X-ray microscopy (STXM) has been used to investigate the spatiotemporal evolution of lithium composition and insertion rates in LiFePO_4_ primary particles (Lim *et al.*, 2016 ▸), but the field of view is relatively small (individual particles) (Bak *et al.*, 2018 ▸). Detailed, temperature-dependent quantification of ionic and electronic circuit elements modeling from electrochemical impedance spectroscopy data was also used that identifies soft-shorts of lithium dendrites (Counihan *et al.*, 2024 ▸). The first neutron and X-ray tomography instrument (NeXT-Grenoble) has been developed that can perform both neutron tomography and X-ray absorption imaging (Tengattini *et al.*, 2020 ▸). *In situ* neutron tomography imaging of lithium electrodeposits in a cycled lithium symmetric cell was performed where the electrochemical cell comprised a natural lithium electrode, a ^6^Li electrode and a deuterated liquid electrolyte (Magnier *et al.*, 2021*a* ▸). The neutron tomography was compared with X-ray tomography images of the same cell acquired both at an X-ray synchrotron beamline and on a laboratory X-ray tomography instrument; the neutron tomography showed good agreement with the X-ray tomography analysis (Magnier *et al.*, 2021*b* ▸).

Early works on X-ray Compton computed tomography (CT) (Gorshkov, 2001 ▸; Gorshkov *et al.*, 2010 ▸; Kupsch *et al.*, 2015 ▸) require reconstruction techniques, for example, filtered back projection or equivalent techniques, to generate a Compton CT slice. In our study, full-field X-ray Compton scattering (XCS) imaging is realized through direct imaging of the sample by collecting the Compton scattering signals perpendicular to the sample along the plane of the incident X-ray beam. XCS can detect lithium at the battery cell level as XCS measures the energy of incoherently scattered photons that have interacted with the valence electrons, including those in the electrode materials (Huang *et al.*, 2022 ▸; Suzuki *et al.*, 2017 ▸). The valence electrons are of interest because they react with lithium ions during battery charging and discharging redox reactions, hence, XCS can quantify the number of lithium ions moving into and being removed from the electrodes.

Previous XCS studies for battery samples used an X-ray pencil beam to perform a raster scan on the samples and used a collimated high-purity germanium detector (HP-Ge) at 90° to the incident beam to measure the intensity of the incoherent scattered X-rays inside the batteries (Suzuki *et al.*, 2018 ▸). However, imaging with pencil beam scanning is time consuming (Suzuki *et al.*, 2019 ▸) which restricts the scan volume, and XCS was mostly used as a standalone technique.

This paper details the design and implementation of a new correlative imaging technique combining two modalities, X-ray Compton scattering and computed tomography (XCS-CT), using two detectors. For XCS imaging, we expand on the previous point-by-point pencil beam by employing a high aspect ratio rectangular cross-section X-ray sheet beam to perform full-field XCS spectra collection across all pixels of the sample plane simultaneously. This reduces the scanning duration of the same coin cell sample area of interest from ∼30 h using a pencil beam to 12 min, enabling us to monitor changes of chemical properties inside batteries during battery charging and discharging. We applied this technique to two types of rechargeable lithium batteries, both assembled in commercially standard coin cell configurations to represent real world applications. Other types of battery cells (*e.g.* pouch cells and rolled cylindrical cells) are also suitable for this characterization technique. For the two types of battery under investigation, one type is a lithium-ion battery (LIB) containing the cathode LiNi_0.8_Mn_0.1_Co_0.1_O_2_ (NMC811) with a bespoke microstructure and a liquid electrolyte at the fully charged state. The other type is a solid-state battery (SSB) using a polyethylene oxide (PEO)-based solid polymer electrolyte (SPE) in place of the conventional flammable liquid electrolyte at charging. In this work, we also present examples of data processing and the results obtained for each type of battery, showing XCS-CT is a powerful technique to study chemical and microstructural properties of the scanned objects.

## 2. Experimental

### 2.1. *In situ* correlative XCS-CT setup

**Figure 1:**
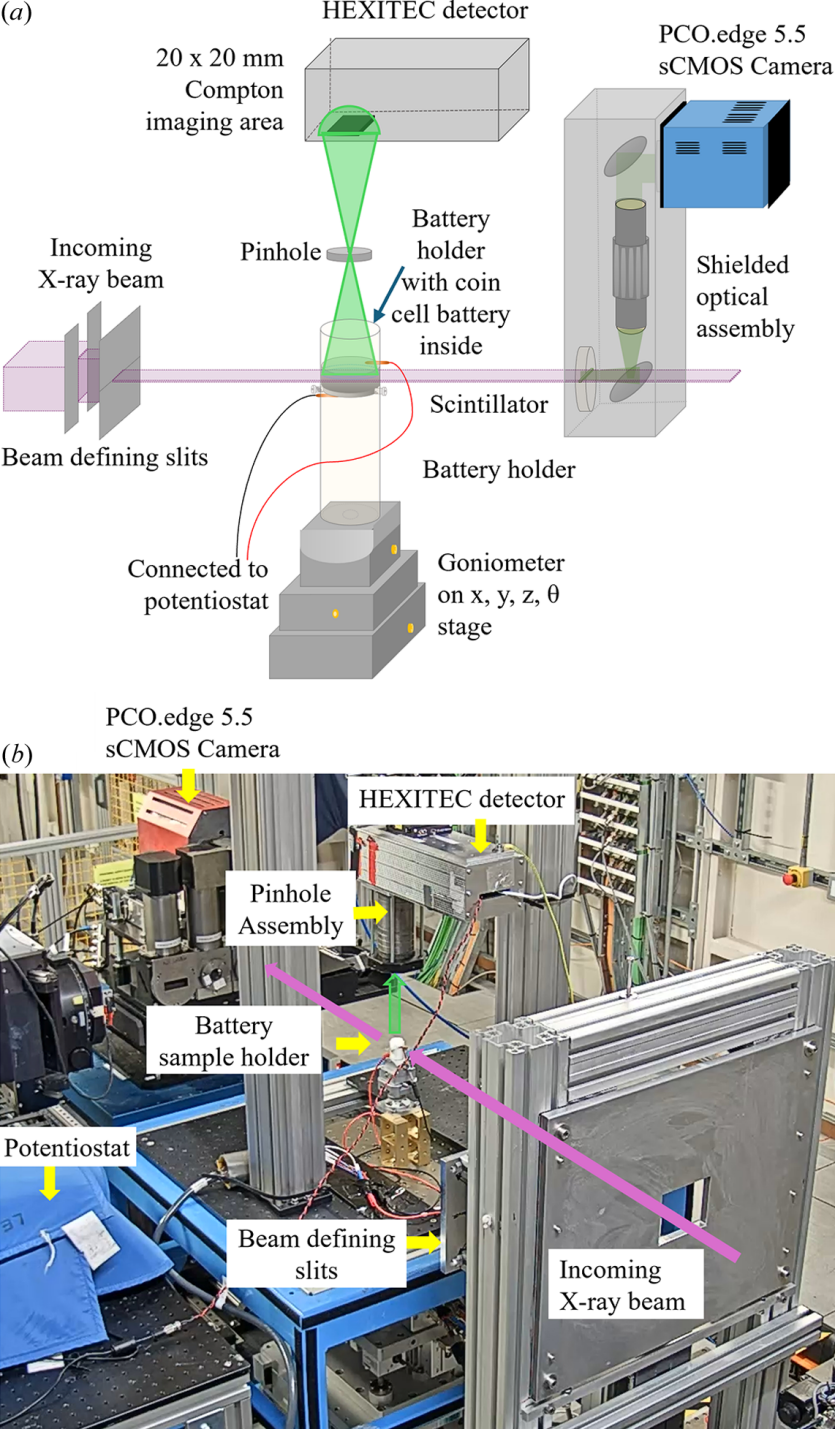
(*a*) Schematic of the XCS-CT experiment setup for the XCS imaging mode where a fan beam was used. For the XCT mode, a box beam was used. (*b*) Photograph of the experimental setup at the beamline.

A schematic and a photograph of the experiment setup are shown in Figs. 1 ▸(*a*) and 1 ▸(*b*), respectively. Both experiments were carried out at the I12 Joint Engineering, Environmental and Processing (JEEP) beamline, Diamond Light Source, UK (Drakopoulos *et al.*, 2015 ▸). A silicon (111) transmission geometry bent Laue double crystal monochromator was used to provide a highly coherent X-ray beam with a photon energy of 114.64 keV measured using a NIST 674b CeO_2_ powder diffraction standard at the beamline (Basham *et al.*, 2015 ▸; Filik *et al.*, 2017 ▸; Hart *et al.*, 2013 ▸).

For the XCT setup, a box beam was used in which the X-rays first interacted with the sample, and then the attenuated X-rays were imaged by the I12 beamline’s standard X-ray imaging detector placed behind the sample, with a 100 µm-thick LYSO-Lu_2_SiO_5_:Ce scintillator lens-coupled to a PCO.Edge 5.5 sCMOS camera. Due to the sample width exceeding the horizontal field of view of the detector at the desired resolutions, an extended horizontal field of view XCT technique with off-centered samples was used (Vo *et al.*, 2021 ▸). Each XCT scan consisted of 3600 projections over 360° with an exposure time of 9 ms per projection. The spatial resolution of the resulting XCT scan was 3.24 µm per pixel.

For XCS imaging, the incident X-ray beam was adjusted to a fan beam (incident beam size 25 mm × 0.25 mm) using horizontal and vertical beam defining slits, where the *xy* plane of the coin cell battery was aligned to be parallel to the fan beam. A high-energy X-ray imaging technology (HEXITEC) detector in a pinhole camera arrangement was positioned at 90° to the incident X-ray beam above the sample to collect scattered photons (Wilson *et al.*, 2015 ▸). The bandwidth of the X-ray beam was set to its maximum value of ∼10^−3^ to maximize the incident flux while being narrower than the energy resolution of the HEXITEC detector (Veale *et al.*, 2018 ▸, 2020 ▸). Scattered X-rays were projected to the HEXITEC detector through a 200 µm-diameter pinhole in a 2 mm-thick tungsten plate with an acceptance angle of 5.7° at ∼160 mm from the sheet X-ray beam. The HEXITEC detector obtained spatially resolved energy spectra for all pixels across the entire plane of a coin cell battery simultaneously, forming an XCS image. The pinhole was placed in an optomechanical system to enable selection of different geometric magnifications by adjusting the distance between the sample, HEXITEC detector and the pinhole. In our work, the distance ratio between the detector to the pinhole and the pinhole to the sample was ∼1:1, achieving an field of view of ∼80 × 80 pixels array or 20 mm × 20 mm with a spatial resolution of 250 µm per pixel (Leung *et al.*, 2023 ▸). 10 mm-thick lead was used to shield the HEXITEC detector from X-rays so it did not collect any that did not originate from the sample.

The HEXITEC detector with a 2 mm-thick CdZnTe material was operated at an acquisition speed of 9 kHz, capturing both the energy and the position of the scattered X-ray photons via an application-specific integrated circuit (ASIC). The resulting data were stored in a data cube of 80 × 80 pixels and 0–200 keV with a step size of 0.2 keV. The average energy resolution (full width at half-maximum, FWHM) was measured to be 0.93 ± 0.19 keV using the 59.54 keV line from an Am-241 sealed source (Koch-Mehrin *et al.*, 2021 ▸). The temperature of the detector was maintained at 18°C with an applied bias voltage of −1000 V. Charge-sharing events occur between pixels when the scattered photons hit more than one pixel on the HEXITEC detector (Veale *et al.*, 2014 ▸), in which the size of the inter-pixel gap is similar to the charge clouds (Veale *et al.*, 2018 ▸). Here, charge-sharing events that spanned more than two neighboring pixels were excluded from the spectral analysis. To obtain XCS imaging of different planes inside the samples, the beamline sample stage was used to move the sample to different vertical positions. Calibration measurements were performed on a completely homogeneous polytetrafluoroethylene (PTFE) disk where a profile without the sample was recorded for flat-field correction to remove signals from the sample holder and other objects.

Rechargeable batteries using bespoke materials and microstructures (Huang & Grant, 2018 ▸) were assembled in standard stainless steel coin cell casings (CR2032) and used for the XCS-CT experiments. The battery samples were connected to an electrochemical potentiostat which set a constant current for charging and discharging the battery at a constant rate of ∼0.1 C. XCT and XCS imaging were performed alternately during battery (dis)charging via remote triggering. The XCT data were collected using standard beamline procedure and the XCS imaging data were recorded into .speciraw files using the *SpecXiDAQ* software (Van Assche *et al.*, 2021 ▸).

### 2.2. Data processing for XCS-CT

Tomographic reconstruction was performed using the *Savu* pipeline, incorporating ring artifact removal and tomographic reconstruction using the *ASTRA* toolboxes (Atwood *et al.*, 2015 ▸; Vo *et al.*, 2018 ▸; Wadeson & Basham, 2016 ▸). This was followed by 3D image processing, quantification and data visualization using a combination of *ImageJ* (Schneider *et al.*, 2012 ▸), *MATLAB* (version 2019b; MathWorks Inc. USA) and *Avizo* (version 2019.2; Thermo Fisher Scientific Corporation, USA), see details in Huang *et al.* (2022 ▸). A 3D median filter with a kernel of 3 × 3 × 3 was used followed by a morphological opening with a radius of 3 voxels, auto-thresholding using the factorization method and then connected component analysis to quantify key metrics components, see the method described in Leung *et al.* (2019 ▸).

For XCS data analysis, the raw data were first converted into a data cube with the dimensions 80 × 80 × number of frames, described in Leung *et al.* (2023 ▸). Then, the data cube underwent dark field, offset, gain and charge-sharing corrections. The pixel energy spectra can be summed together to produce a single global spectrum. To obtain XCS imaging of a specific component/area, we applied an XCT mask of the battery component onto the XCS image. The XCS image was further analyzed using the following method.

The angular distribution (cross sections) of photons scattered from a single free electron is described by the Klein–Nishina formula (Hafiz *et al.*, 2021 ▸), which gives the probability of a scattered photon into the solid-angle element when the incident photon flux is φ_0_. In the energy spectrum of scattered photons, the intensity of the peak d*N* is related to the electron density of the material ρ_e_ excited by the X-ray radiation through equation (1),
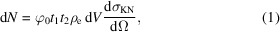
where *t*_1_ is the incident X-ray transmittance from the entrance surface to the probing volume, *t*_2_ is the scattered X-ray transmittance from the probing volume to the exit surface, d*V* is the probing volume and dσ_KN_/dΩ is the Klein–Nishina differential cross section. In the energy spectrum, the most prominent peak is the Compton scattering peak (wherein the incident photon energy is 114.64 keV and the Compton scattering peak is at 94 keV, see results later). A Compton profile was generated at each pixel from the Compton scattering peak through equation (2) (Suzuki *et al.*, 2019 ▸),
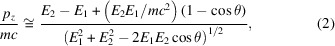
where *p_z_* is a projection of the electron momentum in the core and valence orbitals of the atom or molecule; *E*_1_ and *E*_2_ are the energies of the incident and Compton scattered X-rays, respectively; *m* is the electron mass; *c* is the speed of light; and θ is the scattering angle. A Compton profile is plotted with *p_z_* as the *x* axis and intensity as the *y* axis. The Compton profile is then normalized with the peak located at null electron momentum, *p_z_*. The electron momentum *p_z_* is related to the electron orbital motion speed, and the valence electrons move more slowly than the core electrons, the valence electrons exhibit a lower electron momentum in the range −1 < *p_z_* < 1, whereas the core electrons exhibit a higher electron momentum in the ranges −5 < *p_z_* < −1 and 1 < *p_z_* < 5, as previously estimated by measuring the XCS energy spectra of the cathode at the fully charged and discharged states only (Suzuki *et al.*, 2018 ▸). The valence electrons are those that are transferred between the lithium atom and the cathode material compound for redox reactions during battery charging and discharging; equation (3) is used to calculate electron momentum factor (EMF) which is the ratio of the areas under the Compton profile between the low and high electron momentum. Previous results show that the lithium-ion concentration is directly proportional to the EMF, and the EMF can be used to calibrate the lithium-ion concentration in the cathode materials (Huang *et al.*, 2022 ▸),
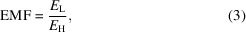
where *E*_L_ and *E*_H_ are the integrals of low and high electron momentum densities in the electron momentum profiles, respectively, as shown by equations (4) and (5) (Leung *et al.*, 2023 ▸; Suzuki *et al.*, 2019 ▸),
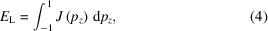

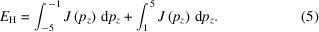


## 3. Results and discussion

**Figure 2:**
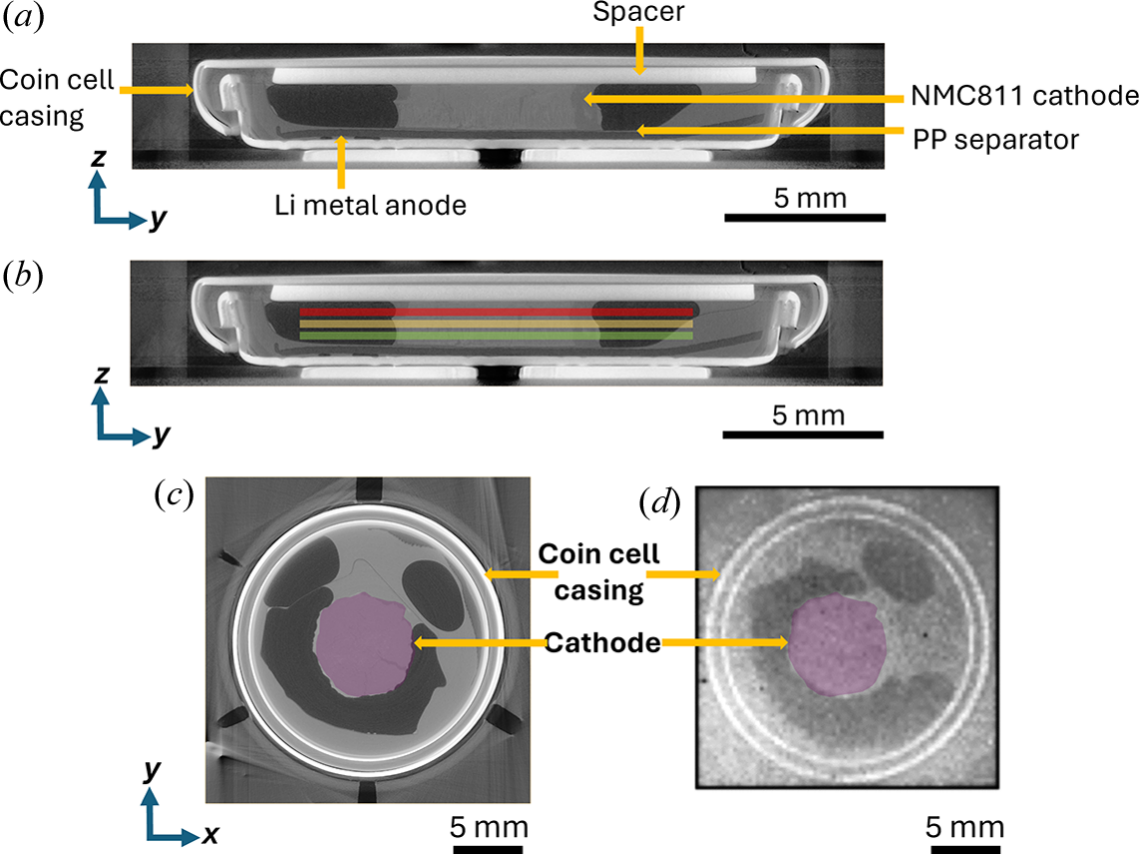
(*a*) A greyscale reconstructed XCT slice in the *yz* plane of a lithium-ion battery assembled in a commercially standard coin cell casing. (*b*) Schematic superimposed onto the XCT slice in (*a*) showing the three different vertical positions of the X-ray sheet beam for XCS imaging. (*c*) Reconstructed XCT and (*d*) XCS imaging slices in the *xy* plane of the battery showing the cathode location (in purple false colour).

Fig. 2 ▸(*a*) displays a greyscale reconstructed XCT slice in the *yz* plane of the first-type lithium-ion battery, showing a thick (∼1 mm) Li_*x*_Ni_0.8_Mn_0.1_Co_0.1_O_2_ (NMC811) cathode made by directional ice templating (Huang *et al.*, 2019 ▸; Huang & Grant, 2018 ▸), a lithium metal anode (∼250 µm), a polypropylene (PP) separator (∼20 µm) and a stainless steel spacer (0.5 mm) inside the coin cell casing. The PP separator was soaked with a liquid electrolyte which occupied some of the remaining volume inside the coin cell.

Fig. 2 ▸(*b*) shows the three vertical depths of the cathode [coloured lines overlaid on the XCT slice in Fig. 2 ▸(*a*)] where XCS imaging was performed. Figs. 2 ▸(*c*)–2 ▸(*d*) are the corresponding XCT and XCS imaging slices in the *xy* plane of the battery in the red region of Fig. 2 ▸(*b*). The cathode is highlighted in purple (false colour) in Figs. 2 ▸(*c*) and 2 ▸(*d*). Although the XCS image is nearly 80 times coarser in pixel resolution than the XCT image, we can still resolve key features in the cathode, the coin cell casing (bright double circles) and bubbles of Ar gas (dark grey region) inside the battery. The bubbles were possibly introduced during cell assembly inside an Ar-filled glovebox.

**Figure 3:**
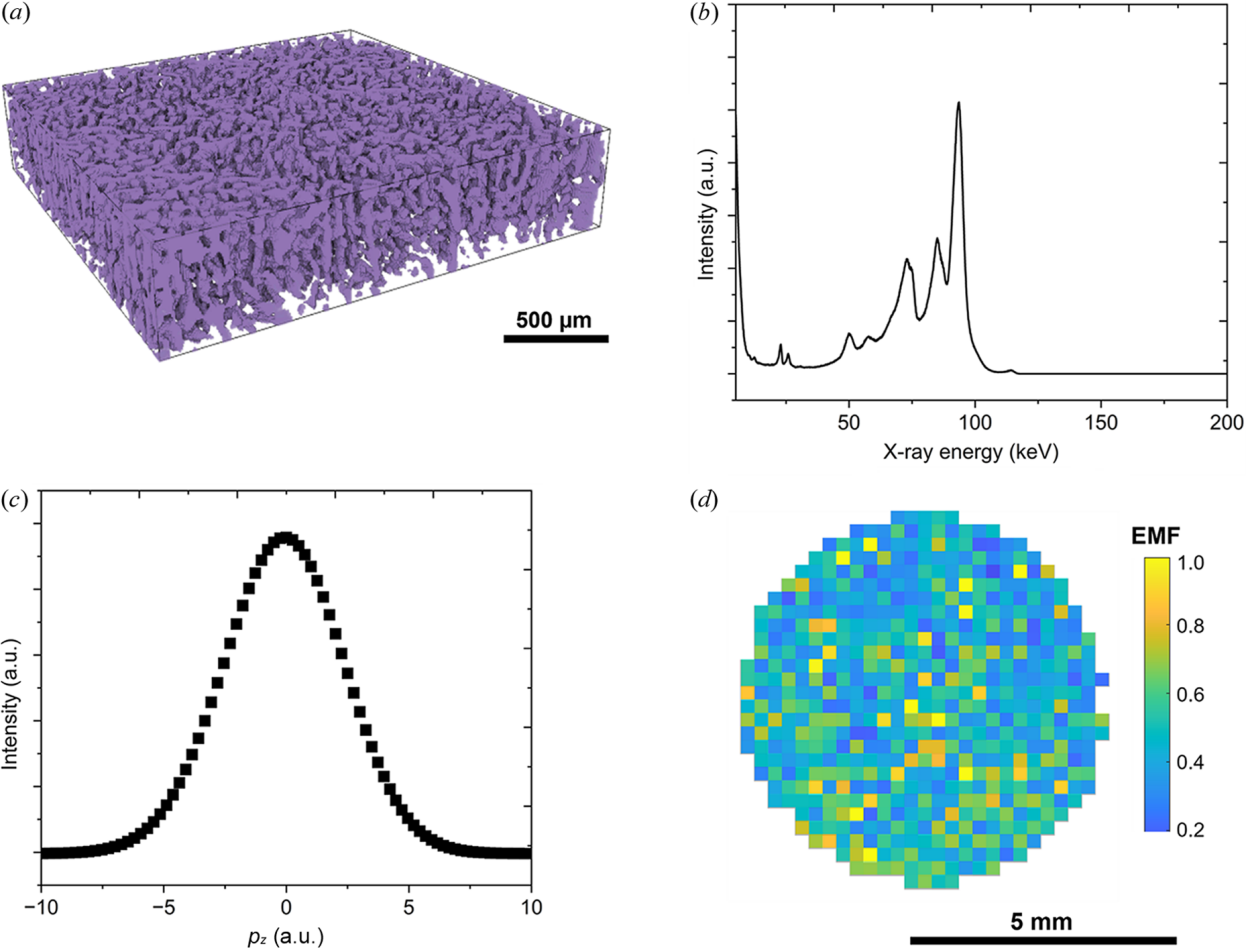
(*a*) 3D volume rendering of the internal structure of the NMC811 cathode inside the lithium battery from the XCT data, showing a porous internal structure. (*b*) Overall energy spectrum obtained by XCS imaging in the top region (furthest from the separator) of the cathode at the fully charged state. (*c*) Corresponding Compton profile obtained from the Compton peak in (*b*). (*d*) EMF mapping across the *xy* plane in the top region of the cathode at the fully charged state from the XCS imaging data.

Fig. 3 ▸(*a*) shows a 3D volume rendering of a porous cathode, where the purple phase is the material phase. The diameter of the internal pores ranged from 5 µm to 20 µm. We applied a mask of the cathode using the XCT image along the *xy* plane onto XCS imaging to show electron momentum within the cathode excluding other components of the cell. Fig. 3 ▸(*b*) shows the overall energy spectrum obtained by summing all pixels in the masked XCS image in the top region of the cathode at the fully charged state. The dominant peak (the Compton peak) occurs at 94 keV which is defined by the inelastically scattered photon energy from the incident photons (Huang *et al.*, 2022 ▸). There is a cluster of peaks at 50–90 keV which belongs to the characteristic X-rays from the tungsten pinhole and lead shielding around the detector, and escape peaks associated with the self-fluorescence of the cadmium and tellurium in the detector material (Thompson & Vaughan, 2001 ▸). Self-fluorescence is also the cause of the peaks at 23–32 keV (Veale *et al.*, 2018 ▸). The low-energy threshold of the detector is ∼5 keV (Kane, 2006 ▸), and a small distinct peak at 115 keV can be attributed to X-ray coherent scattering (Pettifer *et al.*, 2008 ▸).

Fig. 3 ▸(*c*) shows the corresponding overall Compton profile obtained by summing all pixels in one depth region and calculating from the Compton peak at 94 keV using equation (2). The electron momentum factor (EMF) was calculated from the electron momentum density between the valence and the core electrons using equations (3)–(5) (Suzuki *et al.*, 2016 ▸, 2018 ▸), and the overall EMF was 0.65 in the top region of the cathode at the fully charged state. For comparison, the overall EMF in the middle region of the cathode at the fully charged state was calculated to be 0.72. The difference in EMF was caused by different lithium-ion concentrations, *i.e.* a higher lithium-ion concentration in the middle depth region than the top depth region (furthest from the separator). This is because a lithium-ion concentration gradient was developed through the electrode thickness in the thick (∼1 mm) cathode where lithium-ion diffusion was more restricted.

Fig. 3 ▸(*d*) shows mapping of individual EMFs in each pixel along the *xy* plane in the top region of the cathode at the fully charged state. XCS imaging collected an energy spectrum in each pixel that was then converted into a Compton profile, and the EMF of each pixel was obtained and plotted pixel-by-pixel in Fig. 3 ▸(*d*). The electrode exhibited lateral heterogeneity in lithium-ion concentration owing to the internal structure of the cathode [Fig. 3 ▸(*a*)] where the pore phase had higher lithium-ion concentrations as the pores were filled with liquid electrolyte.

**Figure 4:**
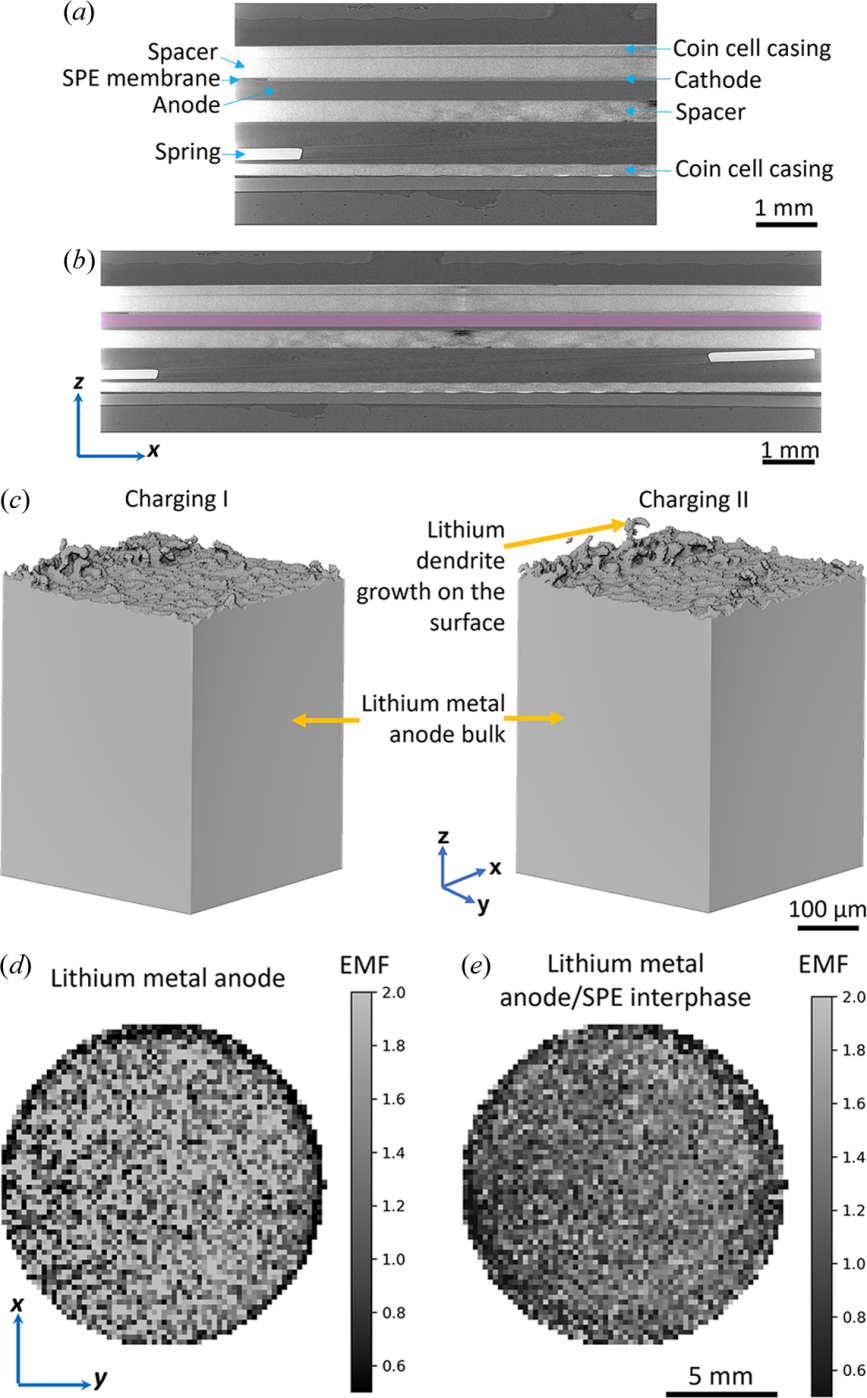
(*a*) Greyscale reconstructed XCT slice in the *yz* plane of a solid-state battery. (*b*) Vertical position (pink area) of the coin cell battery showing one of the positions of the X-ray sheet beam for XCS imaging. (*c*) 3D volume rendering of the interphase on the surface of the lithium metal anode from the solid-state battery coin cell in the middle of charging. XCS images of EMF distributions laterally along the *xy* plane in the middle of charging (*d*) in the lithium metal anode and (*e*) at the interphase between the lithium metal anode and the SPE membrane.

Fig. 4 ▸(*a*) is a greyscale reconstructed XCT slice in the *yz* plane of the second-type solid-state battery fabricated by slurry coating, showing a cathode (65 µm in thickness) containing a mixture of Li_*x*_Ni_0.6_Mn_0.2_Co_0.2_O_2_ (NMC622) material, a polyethylene oxide (PEO) SPE and an LiC_2_F_6_NO_4_S_2_ (LiTFSI) salt, an SPE membrane containing a mixture of PEO and LiTFSI salt (30 µm thick), an Li metal anode (250 µm thick), a stainless steel spring, and two stainless steel spacers (500 µm thick) inside the coin cell casing. Fig. 4 ▸(*b*) shows the location of the X-ray sheet beam for XCS imaging of the lithium metal anode region, the vertical position of the X-ray sheet beam was then moved slightly downwards for XCS imaging of the interphase between the lithium metal anode and the other components. Fig. 4 ▸(*c*) displays time-lapse 3D volume rendering of lithium dendrites growing at the anode–SPE interphase during charging as lithium was deposited heterogeneously on the anode surface. The bulk lithium metal anode was dense and had no obvious microporosity.

Figs. 4 ▸(*d*)–4 ▸(*e*) show the XCS images of EMF distributions laterally along the *xy* plane in the lithium metal anode and at the interphase between the lithium metal anode and the SPE membrane, respectively, in the middle of charging, showing a higher overall EMF in the lithium metal anode at that state. Both EMF maps show a higher EMF in the middle of the anode and SPE membrane compared with the circular edge of the images because the diameter of the anode (16 mm) was larger than that of the cathode (14 mm) and the lithium ions were transported between the anode and the cathode during charging and discharging. Therefore, the middle region of the anode and the SPE membrane in the *xy* plane overlapped with the cathode was more active and contained higher lithium-ion concentrations. In this example, we used EMF to show the variation in lithium-ion concentration across the entire plane of a coin cell battery. The overall EMF was calculated to be 1.7 and 1.1 for the lithium metal anode and the lithium metal anode/SPE interphase, respectively. These results indicate a higher overall lithium-ion concentration in the lithium metal anode than the anode/SPE interphase, *i.e.* slower lithium-ion diffusion kinetics in the solid-state electrolyte than in the lithium anode and this is one of the main challenges for developing solid-state batteries.

## 4. Conclusions

This paper demonstrates that the correlative imaging XCS-CT technique can aid the quantification and mapping of lithium-ion concentration distributions in different regions and components inside rechargeable batteries in a commercially standard coin cell configuration. With XCS-CT, we quantified the number of lithium ions reacting with the valence electrons of electrode materials during battery redox reactions, and we correlated the changes in lithium-ion concentrations with the internal microstructure of the active components. The XCS-CT method can be applied to a wide range of engineering applications to guide future materials development and to study chemical property–structure relationships.
